# Successful Management of Acute Type A Aortic Dissection in a 305 kg Patient Using Two Oxygenators in Parallel

**DOI:** 10.1055/a-2926-8670

**Published:** 2026-08-11

**Authors:** Joseph C. Sweeney, Andrew A. Tully, Gabriel E. Cambronero, Lorrie P. Graf, Matthew D. Lee, Steven C. Eastlack, Adrian L. Lata

**Affiliations:** 1 Department of Cardiothoracic Surgery 12279 Wake Forest University School of MedicineWinston SalemNorth CarolinaUnited States; 2 Department of Perfusion Services 528756 Atrium Health Wake Forest BaptistWinston SalemNorth CarolinaUnited States; 3 Department of Anesthesiology 12279 Wake Forest University School of MedicineWinston SalemNorth CarolinaUnited States

**Keywords:** aorta, aortic valve, dissection

## Abstract

Patient factors can present formidable challenges in emergent aortic surgery. We report successful mechanical aortic root, ascending aorta, and Zone 2 arch replacement under hypothermic circulatory arrest in a 305 kg patient with acute type A aortic dissection. A dual-oxygenator bypass circuit, proactive metabolic management, bilateral fasciotomies, and early renal replacement therapy were critical to a favorable outcome.

## Introduction

Extreme obesity poses unique challenges in emergent cardiac surgery, particularly with acute type A dissection, where technical limitations and poor prognosis often lead to nonoperative management. When taken to the operating room, these patients have been shown to have poorer outcomes. 1 2 3 We report the successful management of a 305 kg patient utilizing innovative perfusion strategies.

## Case Presentation

A 33-year-old man (305 kg, 6'11”, body mass index 68.6 kg/m ^2^ , body surface area 4.23 m ^2^ ) presented with acute type A aortic dissection after being declined transfer by multiple other centers due to size and complexity. At our institution, he underwent composite mechanical aortic root, ascending aorta, and Zone 2 arch replacement using hypothermic circulatory arrest. Lateral bed extenders were used to aid in patient positioning and securement. A cardiopulmonary bypass (CPB) circuit was prepared with dual oxygenators in parallel in preparation for potential physiological limitations.

Innominate artery and right atrial cannulation were performed and CPB commenced. Flows of 6 to 6.6L/min were able to be maintained throughout the duration of CPB. Hemoconcentration was initiated at the onset of CPB to support volume management. The left ventricle was vented through the right superior pulmonary vein. Aortic cross-clamp was applied and both retrograde and ostial Del Nido cardioplegia were administered to achieve diastolic arrest. Operative findings included severe aortic insufficiency on transesophageal echocardiography evaluation, a circumferential tear to the aortic root below the sinotubular junction with dissection down the noncoronary sinus, and circumferential dissection of the aorta around the left coronary ostium.

After 45 minutes of cooling with a nadir of 18.3°C, CPB was discontinued and hypothermic circulatory arrest initiated (96 minutes total; 52 minutes with bilateral antegrade cerebral perfusion at 900 mL/min using near-infrared spectroscopy monitoring for tissue oximetry). The intimal flap continued circumferentially into the arch and around the ostia of the head vessels. Zone 2 aortic arch replacement was performed.

Notably, serum potassium rose from 4.5 mmol/L prior to surgery, to 7.0 mmol/L during hypothermic circulatory arrest, raising concern for rhabdomyolysis. This persisted despite zero-balance ultrafiltration and loop diuretic challenge after cessation of urine output. Lactic acid peaked at 10.6 mmol/L upon resumption of systemic flow. Both potassium and lactic acid gradually improved after restoration of systemic circulation.

With resumption of CPB, aortic root replacement was performed using a 29-mm mechanical aortic valved conduit, employing the modified Cabrol technique for the dissected left coronary button with direct button-to-root reimplantation of the right coronary artery. CPB time was 248 minutes, and aortic cross-clamp time was 210 minutes.

Due to persistent lactic acidosis, elevated potassium, and limited physical exam reliability postoperatively, bilateral four-compartment fasciotomies were performed. Early renal replacement therapy was initiated in the cardiovascular intensive care unit. The patient was extubated on postoperative day 2, and fasciotomy sites were closed on postoperative day 3. He was discharged on postoperative day 14 to acute inpatient rehabilitation and ultimately had complete renal recovery. At his last follow-up visit, he had lost 33 kg, was meeting blood pressure goals with good compliance and medical management, and had returned to work without any deficits. He additionally has been evaluated by the genetics team and is completing a 35-gene panel for evaluation of genetic aortopathy.

## Discussion

This case illustrates that with meticulous planning and innovative perfusion strategies, complex aortic surgery can be successfully performed in patients with extreme obesity. Dual oxygenator CPB support and proactive metabolic management were critical to achieving a favorable outcome, and early initiation of renal replacement therapy may have aided in his recovery.
